# A nonlinear multi-parameter model for predicting floor acceleration amplification across diverse structural systems

**DOI:** 10.1038/s41598-025-26080-3

**Published:** 2025-11-26

**Authors:** Rui Pan, Yalin Yu, Wenbin Zhang, Xiaoshuang Li

**Affiliations:** 1 College of Civil Engineering, Qilu Institute of Technology, Jinan, China; 2 Jinan Rail Transit Group Operation Co., Ltd, Jinan, China

**Keywords:** Floor acceleration amplification, Fundamental period, Strength ratio, Non-structural components, Incremental dynamic analysis, Engineering, Materials science, Natural hazards, Solid Earth sciences

## Abstract

Non-structural components represent a major portion of building investment and experience significant damage during earthquakes, leading to functional loss and economic costs. This study develops a nonlinear multi-parameter model to predict floor acceleration amplification (FAA, defined as the ratio of peak floor acceleration to peak ground acceleration), which is crucial for designing acceleration-sensitive non-structural elements. Incremental Dynamic Analysis was performed on diverse structural systems (reinforced concrete, steel, and steel-concrete composite structures) subjected to scaled ground motions. The research quantified the influence of relative height, fundamental period, strength ratio (representing ductility demand), and structural system type on FAA distribution. The proposed fundamental period, distinct from conventional code approaches relying solely on the relative height. Validated against 59 instrumented building records and compared with numerical simulations and existing models, the model demonstrated superior predictive accuracy across different structural fundamental periods, nonlinear states, and system types. This provides enhanced theoretical understanding and practical support for seismic design, addressing limitations in current code provisions for non-structural components.

## Introduction

In modern buildings, non-structural components not only account for a major portion of the total construction investment—approximately 70–85%—but also tend to experience higher damage rates than structural components during earthquakes. Such damage can result in loss of building functionality, casualties, and substantial economic losses. Therefore, the seismic design of non-structural components is a key aspect in ensuring overall building performance^1^.

**Fig. 1:**
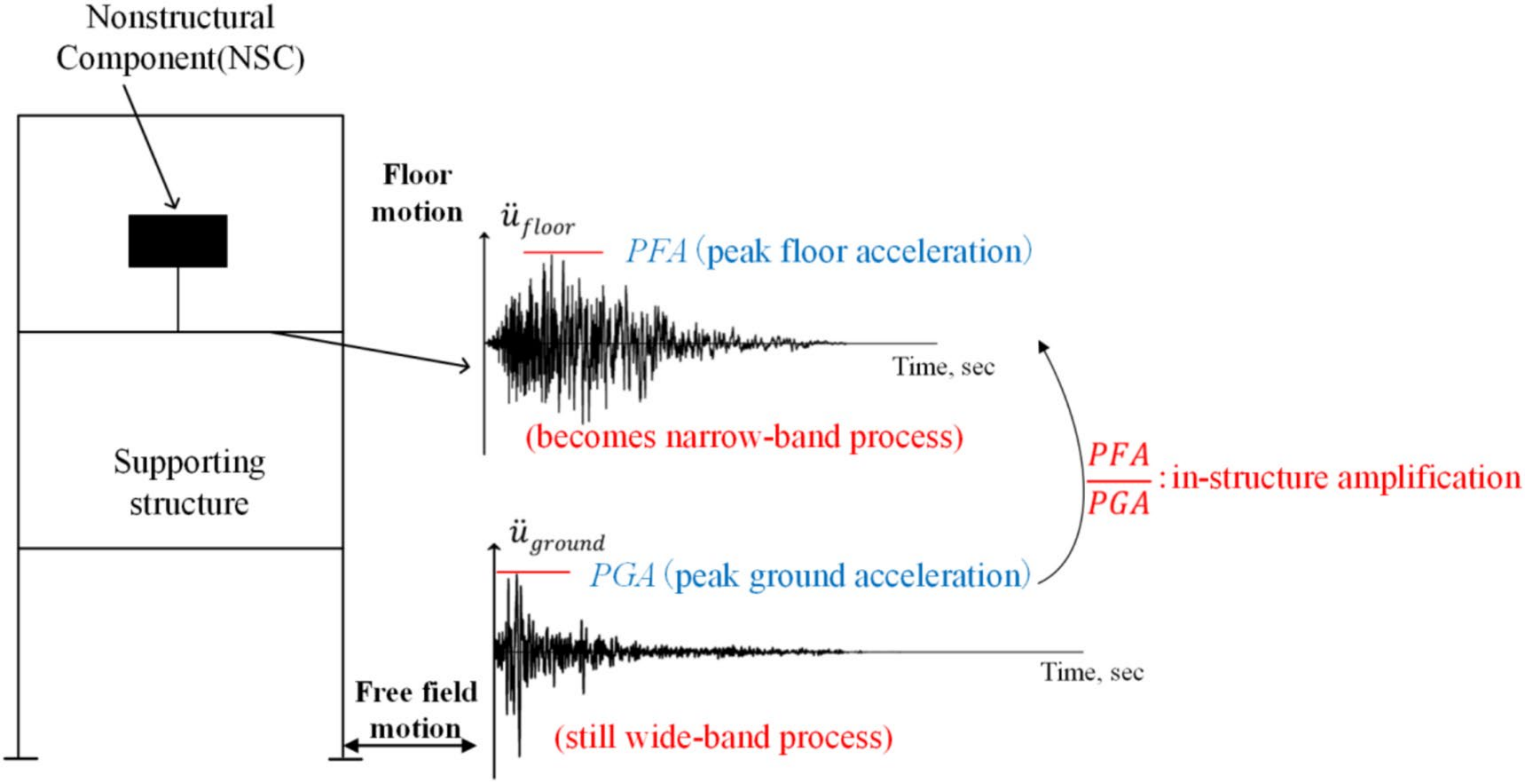
Concept of PFA-PGA amplification mechanism.

Based on the seismic response to earthquake activities, non-structural components can be categorised as either displacement-sensitive or acceleration-sensitive. Displacement-sensitive components, such as curtain walls and piping systems, rely on deformation capacity to accommodate the maximum displacement demands of the main structure. Acceleration-sensitive components, including ceiling systems, fire sprinklers, lighting fixtures, and mechanical equipment, require seismic performance to be ensured through the calculation of equivalent seismic forces^2^. This study focuses on the seismic response mechanisms and design requirements of acceleration-sensitive non-structural components. When estimating the equivalent seismic force acting on such components, one of the key parameters is the Floor Acceleration Amplification (FAA), defined as the ratio of peak floor acceleration (PFA) to peak ground acceleration (PGA). FAA reflects the amplification effect of the structural system on ground acceleration. As illustrated in Fig. 1, floor acceleration can be understood as the result of a dynamic “filtering” process within the structure. Seismic input, expressed as PGA at the free-field ground level, propagates upward through the structure, undergoing dynamic evolution influenced by mass, stiffness, and damping. This process leads to the development of local responses, such as PFA, at various floor levels. Due to the selective amplification of certain frequency components by the structure during transmission, seismic energy tends to be progressively amplified from the ground to higher floors. This frequency-dependent amplification leads to a pronounced acceleration increase within the structure, represented by the FAA^3^.

Current major seismic design codes—such as^4,5,6,7^—commonly estimate FAA using empirical functions of the structural height ratio (*z*/*h*). However, both previous research and earthquake records have shown that this simplified approach may lead to systematic errors in high-rise buildings, flexible systems, and under strong ground motions.

Many international studies have investigated the along-height distribution of FAA in buildings. Early research focused on identifying general trends. Drake and Gillengerten^8^ analysed acceleration records from 150 instrumented buildings in California across 16 earthquake events, showing that FAA exhibits an approximately linear distribution with height. Akhlaghi and Moghadam^9^ conducted nonlinear time-history analyses on five 2D steel frames using 28 ground motions from different site categories, highlighting the influence of both structural properties and site conditions on FAA. Shang et al.^10^examined RC frame models with varying design parameters and vibration periods through elastic and elastoplastic analyses, clarifying the distribution of peak floor acceleration distributions across floors. ? studied a base-isolated structure using shaking table tests, confirming that FAA is sensitive to both structural type and isolation characteristics^12^. further noted that ASCE 7–16 does not adequately envelope recorded FAA responses from instrumented buildings.

Subsequent studies emphasised the effects of structural nonlinearity, ductility, and higher-mode participation on FAA. Fathali and Lizundia^13^, using data from the California Strong Motion Instrumentation Program (CSMIP), reported that FAA tends to be more pronounced under low-intensity earthquakes. Petrone et al.^14^ observed that under strong ground motions, structural nonlinearity and higher mode participation significantly influence the distribution of FAA. Surana et al.^15^ developed a regression model for FAA based on the Incremental Dynamic Analysis (IDA) method, revealing the modulation effect of ductility demand on floor acceleration. Cao et al.^16^ proposed a simplified method to estimate FAA for acceleration-sensitive non-structural components in reinforced concrete (RC) frames designed according to the Chinese seismic code^6^. However, this approach does not account for other types of frame structures^17^, through the analysis of recorded data, found notable differences in PFA amplification between linear and nonlinear structural states. These differences are influenced by both geometric and dynamic parameters, including period shift and mode shape variation. Their study highlights the importance of incorporating structural system type and ductility adjustment mechanisms in the seismic design of non-structural components.

In summary, the study of FAA is of great importance for improving the seismic performance and usage safety of high-rise buildings. Based on recorded data and numerical simulations, researchers have identified four key parameters that determine FAA: structural period, the position of non-structural components, structural nonlinearity, and structural system type. However, current simplified approaches often consider only one or two of these variables. Therefore, a more comprehensive analysis is needed.

To address these limitations, this study designs three types of structures—reinforced concrete frames, steel frames, and steel–concrete composite systems—where buildings of the same structural type share identical plan layouts across different heights, in compliance with^4,6^. Using seven seismic ground motions, IDA are performed under different values of the strength ratio ($R_\mu$) to establish a simplified FAA model. This enables quantification of how structural nonlinear behaviour, fundamental period ($T_1$), structural typology, and non-structural component *z*/*h* collectively govern FAA. Finally, comparative analyses with the^4,6^ codes and existing research provide insights for developing more reliable FAA prediction methodologies.

## Original model design

Seismic records from instrumented buildings in existing databases are generally associated with relatively low levels of ground motion. However, at higher levels of ground motion intensity, buildings are expected to exhibit nonlinear behaviour. To investigate the influence of structural nonlinearity on FAA, this study designs three typical types of frame structures according to China’s seismic design code^6^. These include 5-story, 10-story, and 15-story models of RC frames, steel frames, and steel-concrete composite frames. The selected structural heights and types cover representative low-, medium-, and high-rise building systems, each with different stiffness characteristics, providing good representativeness and applicability for the intended analysis.

The software used for elastoplastic time-history analysis is PKPM^18^. PKPM integrates finite element modeling, automated reinforcement design, and seismic performance evaluation. It strictly follows Chinese design codes, ensuring that the simulation results accurately reflect actual engineering practices. Compared to internationally used platforms such as ETABS and SAP2000, PKPM offers significant advantages in seamless integration with Chinese codes and extensive validation through domestic engineering projects, making it particularly suitable for this study.

All frame structures are assumed to be fixed at the base, with mass concentrated at the floor nodes. The elements used include beam, column, and shell elements. Beam elements contain two nodes (i and j), each with 6 degrees of freedom, including three translational and three rotational components. Column elements are geometrically similar to frame elements, with two nodes (i and j), each having 3 degrees of freedom, corresponding to three translational components (u, v, w). Shell elements are used to simulate floor slabs, shear walls, and other planar components, with a unified “membrane + plate (thick plate)” shell element model. During the analysis, a stiffness degradation method is employed, where the formation of plastic hinges is determined based on the ratio of post-yield to initial sectional stiffness at the member ends.

**Fig. 2:**
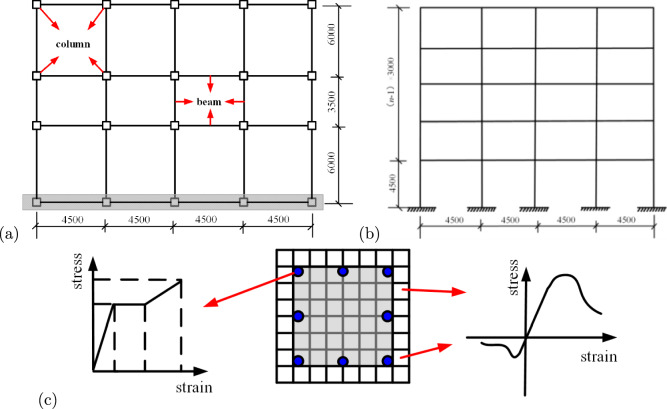
Parameters of reinforced concrete structure models (mm): (**a**) plan; (**b**) elevation, and (**c**) fiber cross-section and materials.

**Fig. 3:**
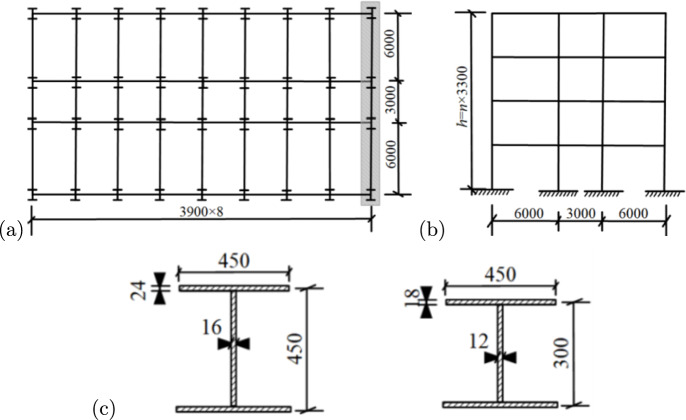
Parameters of steel structure models (mm): (**a**) plan; (**b**) elevation, and (**c**) cross-section dimensions of beams and columns.

**Fig. 4:**
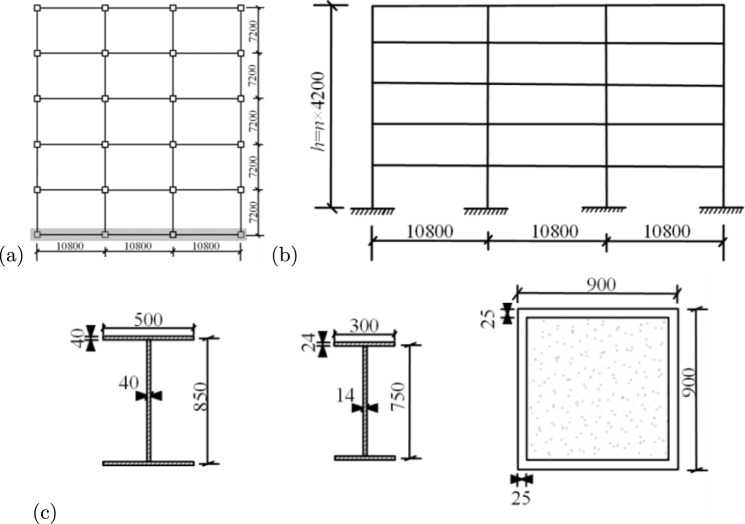
Parameters of steel-concrete composite structure models (mm): (**a**) plan; (**b**) elevation, and (**c**) cross-section dimensions of beams and columns.

In the elastoplastic time-history analysis, appropriate material constitutive relationships are crucial for accurately capturing the structural seismic responses^19^. The constitutive behaviour of steel is modeled using a bilinear elastic-plastic relationship. The elastic modulus is taken as $E_s=200\,\textrm{GPa}$, the yield strength is $f_y=345\,\textrm{MPa}$ according to Q345 steel, and the hardening modulus is assumed to be $E_t=0.01\,E_s$. For concrete, a trilinear constitutive model is adopted to capture its nonlinear characteristics. The peak compressive strength is $\sigma _c=14.3\,\textrm{MPa}$, and the ultimate tensile strength is $\sigma _t=1.43\,\textrm{MPa}$. The ultimate compressive strain is $\varepsilon _u=0.0035$, and the ultimate tensile strain is $\varepsilon _t=0.0015$. The strain corresponding to peak compressive stress is taken as $\varepsilon _c=0.002$. To accurately model the structural behaviour under strong seismic motions, the elastoplastic time-history analysis simultaneously considers material nonlinearity and geometric nonlinearity, including the $P-\Delta$ effect. The $P-\Delta$ effect, which accounts for the interaction between large displacements and internal forces, is particularly important in high-rise buildings and has been included in all analyses using PKPM’s stiffness degradation method.

To ensure accuracy and consistency with real-world engineering practices, all material properties are based on China’s national standards for construction materials. For the RC frames, concrete and reinforcement parameters are selected in accordance with the “General Code for Concrete Structures”^6^, ensuring that the beam and column designs reflect typical structural configurations used in high-rise buildings. The concrete for beams is C30 (characteristic compressive strength 30 MPa). For the columns on the first and second floors, C40 concrete (40 MPa) is used, while C30 concrete (30 MPa) is used for columns on the third floor and above. The reinforcement consists of HRB400 steel bars (nominal yield strength 400 MPa). In the steel frames, the steel selection is consistent with the “Code for Design of Steel Structures”^20^ and provides high strength and ductility under seismic loads. The steel frame uses I-shaped sections made of Q345 steel (nominal yield strength 345 MPa). The steel-concrete composite structures are designed to provide enhanced performance by combining the high strength of steel with the stiffness and energy dissipation capacity of concrete. For the steel-concrete composite structures, the concrete grades used are C60 (60 MPa) and C50 (50 MPa), while the steel tubes are uniformly made of Q345 steel (345 MPa). The modeling parameters for the RC frames, steel frames, and steel-concrete composite frames are shown in Figs. 2, 3, and 4, respectively. The detailed dimensions of beams and columns, as well as the fundamental periods ($T_1$) of the structures, are summarised in Table 1. The $T_1$ values for each structure are considered within the range of 0.5–3.0 s.

**Table 1:** Dimensions and structural period of structural beam and column components.

Structural type	Stories		Column ($\textit{b}\times h$) /mm	Beam ($b\times h$)/mm		$T_1$ (s)
				x-direction	y-direction	
RC frame	n = 5	1$\sim$2	$750\times 750$	$350\times 700$	$350 \times 700$	0.66
		$3\sim 5$	650$\times$ 650			
	n = 10	1$\sim$2	750$\times$ 750	$350 \times 700$	$350 \times 700$	1.37
		$3 \sim 10$	650$\times$ 650			
	n = 15	1$\sim$5	900$\times$ 900	$350\times 700$	$350\times 700$	2.04
		6$\sim$15	750$\times$ 750			
Steel frame	n = 5	1$\sim$2	$450\times 450 \times 16\times 24$	$500\times 350\times 14\times 20$	$500\times 350\times 14\times 20$	0.78
		$$3\sim 5$$				
	n = 10	$1\sim 2$	$450\times 450\times 16\times 24$	$500\times 350\times 14\times 20$	$500\times 350\times 14\times 20$	1.65
		3$\sim 10$				
	n = 15	$1 \sim 5$	$500\times 500\times 20\times 30$	$500\times 350\times 14\times 20$	$500\times 350\times 14\times 20$	2.57
		$6\sim 15$	$450\times 450\times 18\times 28$			
Steel-concrete composite frame	n =5	$1\sim 2$	$900\times 900$	$850\times 500\times 40\times 40$	$750\times 300\times 14\times 24$	0.86
		$$3\sim 5$$				
	n = 10	$1\sim 2$	$900\times 900$	$850\times 500\times 40\times 40$	$750\times 300\times 14\times 24$	1.86
		$$3 \sim 10$$				
	n = 15	$1 \sim 5$	$1000\times 1000$	$850\times 500\times 40\times 40$	$750\times 300\times 14\times 24$	2.89
		$6 \sim 15$	$900\times 900$			

## Ground motion selection and IDA method

### Ground motion selection

The ground motion records used in this study were selected from the PEER database, based on their ability to effectively excite the nonlinear response of frame structures. To ensure that the selected ground motions are representative of the excitation of nonlinear behaviour in frame structures, three criteria were followed^21^: (1) The distance from the recording station to the nearest fault is less than 20 km. Near-field ground motions within this range are likely to contain high-energy short-period components, which more easily drive the frame structures into a nonlinear state. (2) The earthquake magnitude is greater than or equal to 6.0, providing sufficient seismic input strength to trigger the nonlinear response of the structure. (3) The site conditions correspond to hard soil or rock, where the seismic wave propagation characteristics are relatively stable. This minimises the interference of site condition differences on both the ground motion and structural response, allowing for a focused analysis of the structure’s inherent nonlinear behaviour. Ultimately, seven ground motion records were selected from the PEER strong-motion database, and their associated parameters are provided in Table 2.

**Table 2:** 7 Ground Motion Records.

Event	Station	$M_0$ (N$\cdot$m)	Site Type	$M_{wg}$	$R_{jb}$ (km)	PGA (g)
Loma Prieta, 1989	Gilroy Array #3	$2.85\times 10^{19}$	D	6.8	12.23	0.54
Kocaeli-Turkey, 1999	Arcelik	$2.82\times 10^{20*}$	C	7.5	10.56	0.13
Cape Mendocino, 1992	Centerville Beach-Naval Fac	$3.1\times 10^{19}$	C	6.8	16.44	0.32
Imperial Valley-06, 1979	Delta	$6.31\times 10^{18*}$	D	6.3	20.0	0.22
Duzce-Turkey, 1999	Bolu	$7.08\times 10^{19*}$	D	7.1	12.0	0.74
Kobe-Japan, 1995	Shin-Osaka	$2.51\times 10^{19*}$	D	6.7	19.1	0.20
Superstition Hills- 02, 1987	Poe Road	$9.4\times 10^{18}$	D	6.4	11.1	0.29

^a^$M_0$ represents the seismic moment, a physical quantity used to describe the size of an earthquake, with the unit $\hbox {N}\cdot \hbox {m}$. It reflects the energy released during the earthquake rupture process.

^b^$M_{wg}$ is the generalised seismic moment magnitude, calculated based on the formula $M_{wg}=(logM_0)/1.36-12.68$ proposed by^22^. This magnitude scale is designed to be more consistent globally and avoid the saturation issues that traditional magnitude scales face for moderate - to - small earthquakes.

^c^The asterisk (*) next to some M_0_ values indicates that these seismic moment values are derived from specific models or indirect estimations, as per^22^. Compared to directly measured seismic moment data from primary sources, they may have relatively higher uncertainties.

**Fig. 5:**
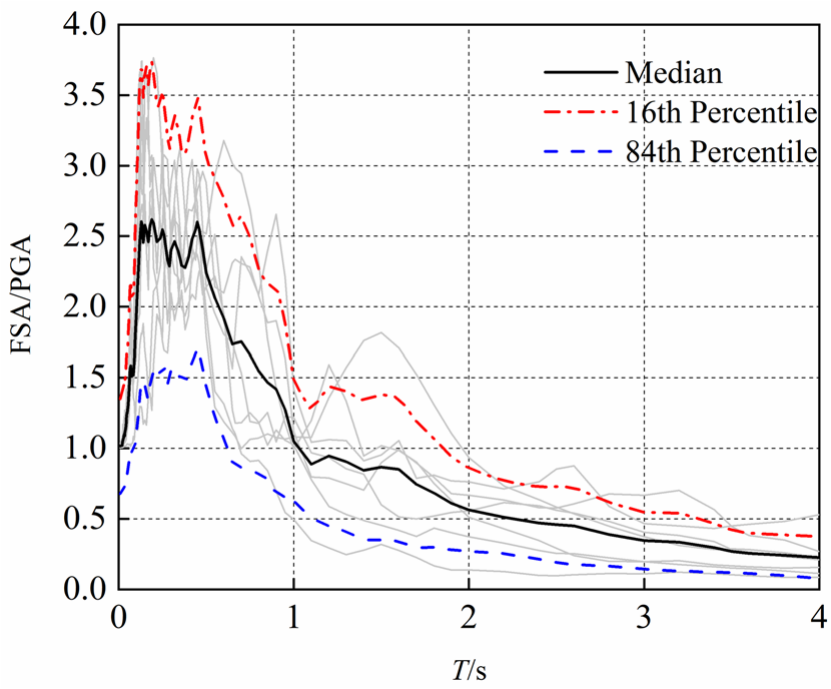
5%-damped acceleration response spectra for the 7 ground motions.

Figure 5 shows the 5% damping acceleration response spectra for the selected seven ground motions, including the response spectra for each individual record, as well as the mean spectrum, the $16^{\textrm{th}}$ percentile spectrum (corresponding to the mean minus one standard deviation), and the $84^{\textrm{th}}$ percentile spectrum (corresponding to the mean plus one standard deviation). It is evident that there is a significant numerical gap between the $16^{\textrm{th}}$ percentile, median, and $84^{\textrm{th}}$ percentile spectra, clearly illustrating the dispersion characteristics of the seven ground motion response spectra across the full period range (*T*). This variation reflects the differences in the dynamic excitation caused by the different ground motions. Such variability in the ground motions will directly affect the calculation results of FAA, highlighting the necessity of considering the differences between ground motion records in FAA analysis. Moreover, this provides the basis for selecting ground motions for subsequent incremental dynamic analysis (IDA), supporting the evaluation of the dynamic response patterns and seismic performance variability of both the frame structure and non-structural components during IDA.

### IDA method

To quantify the effect of seismic intensity on the Floor Acceleration Amplification (FAA) factor, this study adopts the IDA method proposed by^23^. Compared to a single dynamic time history analysis, IDA amplifies the intensity of ground motion records incrementally, generating a series of nonlinear time history analysis results at different intensity levels, thereby enabling continuous assessment of the structure’s dynamic response across multiple earthquake intensity levels.

#### IDA analysis parameters and intensity scaling

The elastic spectral acceleration $S_a(T_1, 5\%)$ at the fundamental period $T_1$ of the frame structure, with a 5% damping ratio, is used as the benchmark parameter for seismic intensity. For the selected seven ground motions, intensity scaling is performed to induce varying degrees of nonlinear behaviour in the structure. The ductility demand coefficient $R_\mu$, defined by^15^, characterises the structural nonlinear strength level. The physical meaning of $R_\mu$ is “the ratio of actual structural ductility to yield ductility”, which directly reflects the degree to which the structure enters the inelastic state. The strength ratio is defined as:

1 $$\begin{aligned} R_\mu = \frac{S_a(T_1, 5\%)}{S_{ay}} \end{aligned}$$

where $S_a(T_1, 5\%)$ is the elastic spectral acceleration at the $T_1$ with 5% damping, and $S_{ay}$ is the yield spectral acceleration.

All building models undergo bidirectional incremental dynamic analysis using seven ground motion records (analysis directions are along the primary lateral and vertical directions, X and Y axes). The value of $R_\mu$ ranges from 0.5 to 3.5, with an interval of 0.5 (seven intensity levels in total). Specifically, $R_\mu = 0.5$ corresponds to a fully elastic state (no significant plastic deformation), $R_\mu = 1.0$ marks the onset of significant inelastic behaviour (formation of plastic hinges), and $R_\mu = 3.5$ represents the maximum ductility demand set to avoid collapse-type failure.

To comprehensively assess the distribution of FAA along the structural height, FAA values are extracted at five different relative heights $z/h$ (where $z$ is the floor height and $h$ is the total structure height), namely $z/h = 0.2, 0.4, 0.6, 0.8, 1.0$, in order to capture the continuous variation of FAA.

#### Capacity spectrum conversion and yield parameters determination

As the IDA method requires clear identification of the spectral acceleration reference value at the structure’s yield point, this study uses the Capacity Spectrum Method (CSM) to convert the Multi-Degree-of-Freedom (MDOF) frame structure into an equivalent Single-Degree-of-Freedom (SDOF) system. The specific conversion process is as follows:

(1) Conversion of the Push-over Curve to the Capacity Spectrum

First, a static push-over analysis was conducted on the frame structure. To account for bidirectional seismic effects within a simplified unidirectional pushover framework, a strategy compliant with the ‘bidirectional seismic action effect combination’ principle in^6^ (Clause 5.2.3) was adopted. The primary response direction (X-direction) was subjected to an inverted triangular lateral load pattern. The effect of seismic excitation in the secondary direction (Y-direction) was considered by amplifying the seismic demand in the X-direction. This was achieved by defining a target base shear for the pushover analysis. First, a reference base shear, $V_{b,x}$, associated with the inverted triangular load pattern in the X-direction, was determined. This reference value was then amplified by a factor $\eta$ to obtain the target base shear:

2 $$\begin{aligned} V_{b,\text {applied}} = \eta \cdot V_{b,x} \end{aligned}$$

The amplification factor $\eta$ was derived from the code’s SRSS combination rule:

3 $$\begin{aligned} \eta = \sqrt{1 + \left( 0.85\frac{S_y}{S_x}\right) ^2} \end{aligned}$$

Assuming that, in the elastic range, the seismic effect is proportional to lateral stiffness $\left( \frac{S_y}{S_x} \approx \frac{K_y}{K_x} \right)$, $\eta$ was determined based on the stiffness ratio $\left( \frac{K_x}{K_y} \right)$ as follows:

1. When $$\frac{K_x}{K_y} \approx 1.0$$; $$\eta = 1.20$$ to $$1.25$$.
2. When $$2.0 \le \frac{K_x}{K_y} < 3.0$$; $$\eta = 1.10$$ to $$1.15$$.
3. When $$1.5 \le \frac{K_x}{K_y} < 2.0$$; $$\eta = 1.15$$ to $$1.20$$.
4. When $$\frac{K_x}{K_y} \ge 3.0$$; $$\eta = 1.08$$ to $$1.10$$.

The magnitude of the inverted triangular load was then scaled during the nonlinear pushover analysis until the resulting base shear equalled $V_{b,\text {applied}}$. The complete base shear $(V_b)$ versus top displacement $(u_n)$ curve up to this target point was recorded.

(2) MDOF-SDOF Equivalent Conversion

To achieve the equivalent simplification of the MDOF frame structure to an SDOF system, the push-over curve’s base shear $V_b$ and top displacement $u_n$ are scaled based on the first mode parameters of the structure (mode participation factor $\Gamma _1$ and effective mass $M_1^*$). The specific conversion formula is as follows^24^:

4 $$\begin{aligned} S_{a}=\frac{V_{b}}{M_{1}^{*}},\qquad S_{d}=\frac{U_{n}}{\Gamma _{1} \phi _{n 1}^{2}} \end{aligned}$$

**Fig. 6:**
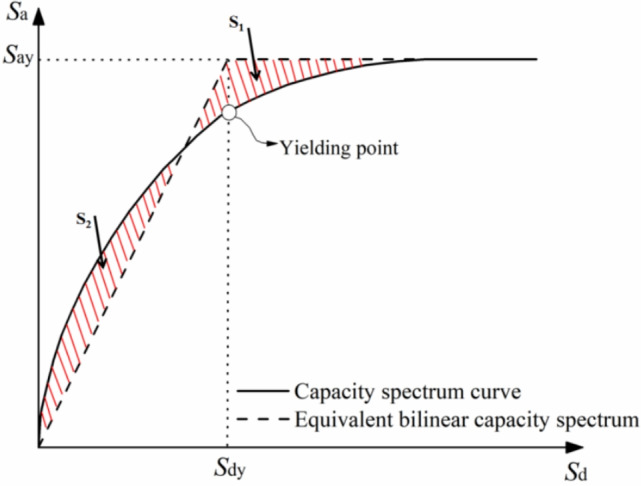
Idealized bilinear equivalent capacity spectrum of the main response direction (including bidirectional effect integration).

Where $\Gamma _1$, $M_1^*$ are the participation factor and effective mass of the first mode, respectively, $V_b$, $U_n$ are the base shear force and vertex displacement, respectively. $S_a$ is the spectral acceleration and $S_d$ is the spectral displacement.

5 $$\begin{aligned} M_1^*=\frac{\left( \sum _{i=1}^nm_i\phi _{i1}\right) ^2}{\sum _{i=1}^nm_i\phi _{i1}^2},\qquad \Gamma _1=\frac{\sum _{i=1}^nm_i\phi _{i1}}{\sum _{i=1}^nm_i\phi _{i1}^2} \end{aligned}$$

where $m_i$ is the mass of the i-layer particle, $\phi _{i1}$ is the amplitude of the i-layer particle under the first vibration mode ; *n* is the number of floors. This conversion step simplifies the MDOF system to an SDOF system. This conversion translates the structural capacity from the $V_b-U_n$ domain of the MDOF system to the $S_a-S_d$ domain of the equivalent SDOF system, and the simplified capacity spectrum is shown in Fig. 6.

(3) Determination of Yield Point and Yield Spectral Acceleration

Following the equivalence principle of energy proposed by^25^, which states that the structural elastoplastic energy dissipation equals the equivalent elastic energy dissipation ($S_1 = S_2$), the capacity spectrum curve is idealised as a bilinear equivalent capacity spectrum. From this, the yield spectral displacement $S_{dy}$ is determined. The spectral acceleration corresponding to $S_{dy}$ on the bilinear curve is the yield spectral acceleration $S_{ay}$, as shown in Fig. 6, which illustrates the idealised bilinear capacity spectrum (Note: Figure 6 presents the equivalent capacity spectrum of the main response direction, with lateral load applied in the main direction and bidirectional effects integrated).

(4) Ground Motion Scaling Formula

For different target ductility demand coefficients $R_\mu$, the spectral acceleration $S_a(T_1, 5\%)$ corresponding to each $R_\mu$ is first determined using the bilinear capacity spectrum. Then, ground motion acceleration scaling is performed based on the original characteristics of the ground motion. Specifically, the original spectral acceleration $S_a(T_1, 5\%)$ of each ground motion at the fundamental period $T_1$ is taken as the baseline, and the acceleration time history $A(t)$ of the original ground motion is linearly scaled using the following formula to obtain the scaled acceleration time history $A'(t)$:

6 $$\begin{aligned} A'(t) = \frac{S_a'(T_1, 5\%)}{S_a(T_1, 5\%)} \times A(t) \end{aligned}$$

where $A'(t)$ is the scaled acceleration, $A(t)$ is the original acceleration, $S_a(T_1, 5\%)$ is the spectral acceleration of the building at the fundamental period $T_1$ before scaling, and $S'_a(T_1, 5\%)$ is the target spectral acceleration after scaling.

## Floor acceleration amplification principles and analysis of representative building floors

To investigate the floor acceleration response characteristics of multi-storey frame structures under strong ground motions, this study conducts nonlinear IDA on three types of structural systems—RC frames, steel frames, and steel–concrete composite frames—using seven sets of earthquake records. The influence of $z/h$, $T_1$, $R_\mu$, and structural system type on FAA is examined. In addition, the acceleration amplification behaviour at critical locations, such as the top storey, is further discussed.

### FAA influencing parameters analysis

#### Effect of *z*/*h* on FAA

**Fig. 7:**
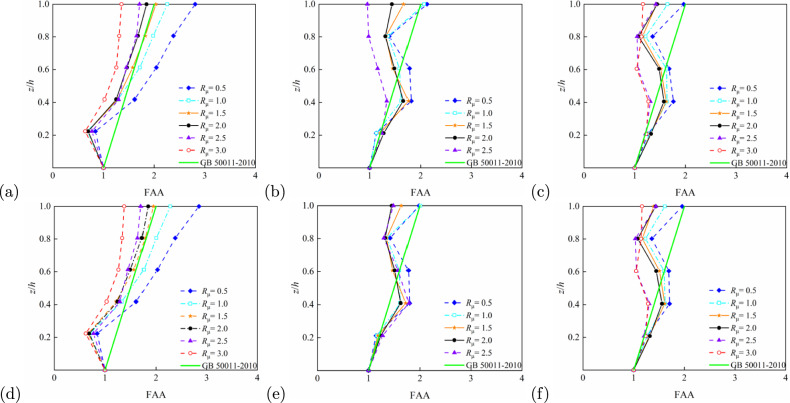
FAA demands as obtained from IDA for different strength ratio of reinforced concrete structures: (**a**) 5-storey, longitudinal ($T_1=0.6624$ s); (**b**) 10-storey, longitudinal ($T_1=1.3652$ s); (**c**) 15-storey, longitudinal ($T_1=2.0369$ s); (**d**) 5-storey, horizontal ($T_1=0.6384$ s); (**e**) 10-storey, horizontal ($T_1=1.3083$,s), and (**f**) 15-storey, horizontal ($T_1=1.9343$ s).

**Fig. 8:**
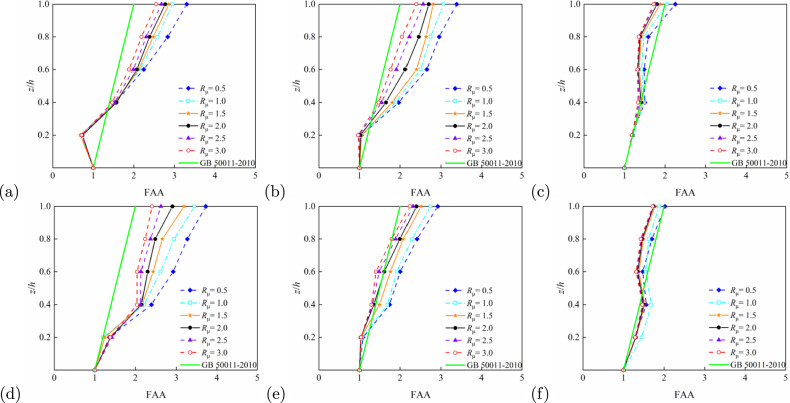
FAA demands as obtained from IDA for different strength ratio of steel structures: (**a**) 5-storey, longitudinal ($T_1=0.7798$ s); (**b**) 10-storey, longitudinal ($T_1=1.6500$ s); (**c**) 15-storey, longitudinal ($T_1=2.5733$ s); (**d**) 5-storey, horizontal ($T_1=0.9033$ s); (**e**) 10-storey, horizontal ($T_1=1.8724,\hbox {s}$), and (**f**) 15-storey, horizontal ($T_1=2.6973\,\hbox {s}$).

**Fig. 9:**
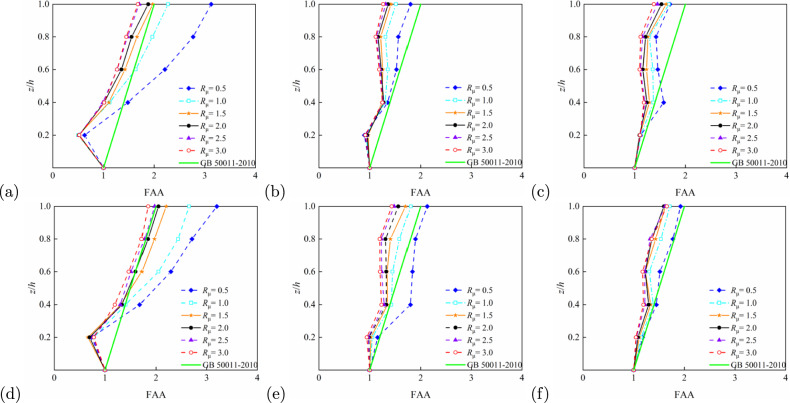
FAA demands as obtained from IDA for different strength ratio of steel-concrete composite structures: (**a**) 5-storey, longitudinal ($T_1=0.8620$ s); (**b**) 10-storey, longitudinal ($T_1=1.8586$ s); (**c**) 15-storey, longitudinal ($T_1=2.8889$ s); (**d**) 5-storey, horizontal ($T_1=0.6635$,s); (**e**) 10-storey, horizontal ($T_1=1.3785$,s), and (**f**) 15-storey, horizontal ($T_1=2.1184$ s).

Relative height, (*z*/*h*), is a significant geometric parameter influencing the floor acceleration amplification effect. Figures 7 and 8 demonstrate that the FAA values generally increase with *z*/*h*, reaching their maximum at the structural top ($z/h = 1$). This trend remains consistent across different structural types and period conditions. For example, in the 5-storey RC frame structure ($T_1$ = 0.6624 s) shown in Fig. 7a, FAA values rise rapidly with floor height, with particularly pronounced amplification at the top level. Figure 7b,c illustrate 10- and 15-storey structures, respectively; although the slope of FAA increase moderates, significant amplification persists at the top. Similar patterns emerge for the steel frames (Fig. 8), notably in panels Fig. 8b,e, where top-level amplification remains noteworthy despite milder FAA variations. Steel-concrete composite structures exhibit analogous behaviour: Figure 9a (5-storey, longitudinal) and d (5-storey, transverse) confirm enhanced FAA with height, indicating acceleration amplification potential in upper zones. Overall, higher *z*/*h* correlates with more substantial floor acceleration amplification, with peak effects consistently observed at the top level.

#### Effect of $R_\mu$ on FAA

The strength ratio, $R_\mu$, of a structure reflects its ductility and seismic capacity, significantly influencing FAA. Multiple comparative results from Figs. 7, 8 and 9 demonstrate that FAA peaks at low $R_\mu$ (e.g. $R_\mu = 0.5$) and gradually decreases with increasing $R_\mu$. In the 5-storey structure shown in Fig. 7a, FAA reaches its maximum value at the top level when $R_\mu = 0.5$, exhibiting the most pronounced amplification effect. Figure 7b,c reveal a clear decreasing trend in FAA as the $R_\mu$ increases from 0.5 to 2.0. This pattern is further confirmed by results in Figs. 8a and 9b, particularly for steel-concrete composite structures where the FAA reduction is more significant, indicating superior strength control capability. Consequently, lower structural $R_\mu$ correspond to larger FAA values, while higher ratios effectively reduce acceleration amplification, demonstrating the critical role of structural ductility and energy dissipation capacity in seismic response control.

#### Effect of $T_1$ on FAA

The fundamental period, $T_1$, as a core parameter characterising structural dynamic behaviour, exerts significant influence on the distribution of FAA. Analysis of various structural heights and period conditions in Figs. 7, 8 and 9 reveals that with increasing period length, the FAA variation pattern gradually stabilises and top-level amplification effects become less pronounced. For instance, in the 5-storey structure ($T_1 = 0.6624$ s) shown in Fig. 7, FAA exhibits a sharp increase with height, whereas in the 10-storey ($T_1 = 1.3652$ s) and 15-storey ($T_1 = 2.0369$ s) structures, the FAA progression stabilises with reduced amplification at the top level. Longer-period steel structures in Fig. 8c,e similarly display relatively uniform acceleration distributions, demonstrating the stability advantages of flexible systems during seismic events. Overall, shorter-period rigid structures tend to produce greater floor acceleration amplification, while longer-period flexible structures exhibit moderated acceleration responses in upper regions due to different energy transfer mechanisms.

#### Effect of structural type on FAA

Based on the roof FAA values summarised in Table 3, the influence of structural type on FAA can be further elucidated. Buildings with 5, 10, and 15 stories were selected, and the FAA performance of reinforced concrete (RC) frames, steel frames, and steel–concrete composite structures was compared, revealing significant differences among the three structural types.

For 5-story buildings, when $R_\mu = 0.5$, the roof FAA of the RC frame is 2.81 in the longitudinal direction and 2.85 in the transverse direction; for the steel frame, the corresponding values are 3.30 and 3.73; for the steel–concrete composite structure, 3.13 longitudinally and 3.20 transversely. At this intensity, the FAA of the RC frame is lower than that of the steel frame and steel–concrete composite structure. As $R_\mu$ increases to 3.0, the RC frame’s FAA decreases to 1.36 longitudinally and 1.38 transversely, whereas the steel frame shows values of 2.55 and 2.40, and the steel–concrete composite structure shows 1.68 and 1.84. The RC frame exhibits a more pronounced reduction in FAA, reflecting its high sensitivity to ductility demand.

For 10-story buildings, at $R_\mu = 0.5$, the RC frame has roof FAA values of 2.12 longitudinally and 1.98 transversely; the steel frame shows 3.39 and 2.92; and the steel–concrete composite structure has 1.80 and 2.13. When $R_\mu = 2.5$, the RC frame’s FAA decreases sharply to 0.96 longitudinally and 1.49 transversely; the steel frame retains 2.56 and 2.31; and the steel–concrete composite structure shows 1.32 and 1.48. This rapid decline indicates the limited ductility and energy dissipation capacity of the RC frame, making it highly sensitive to variations in $R_\mu$ and relative height $z/h$.

For 15-story buildings, at $R_\mu = 0.5$, the RC frame exhibits FAA values of 1.97 longitudinally and 1.96 transversely; the steel frame, 2.25 and 2.02; and the steel–concrete composite structure, 1.70 and 1.92. When $R_\mu = 3.0$, the RC frame’s FAA decreases to 1.16 longitudinally and 1.17 transversely; the steel frame shows 1.72 and 1.72; and the steel–concrete composite structure, 1.38 and 1.65. The steel frame demonstrates more stable FAA variations across different $R_\mu$ levels, highlighting its ductility advantage, which effectively mitigates acceleration amplification and distributes seismic demand more uniformly. The steel–concrete composite structure generally exhibits lower FAA than the RC frame and presents a smoother decline with increasing $R_\mu$, indicating that the composite action of steel and concrete enhances both stiffness and ductility, achieving balanced performance in FAA control.

In summary, under the same story height, the RC frame is more sensitive to reductions in $R_\mu$ and variations in relative height $z/h$, resulting in weaker FAA control. The steel frame, benefiting from its superior ductility, exhibits the most robust FAA performance. The steel–concrete composite structure achieves a good balance between acceleration mitigation and structural stability.

### FAA characteristics at the structural top

To further clarify the effects of structural typology, $R_\mu$, and $T_1$ on the FAA at roof, this study compiles the roof-level FAA values for three structural frame types under varying $R_\mu$ (Table 3), and constructs a three-dimensional relationship diagram between FAA, $T_1$, and $R_\mu$ (Fig. 9). For validation, the results are compared against current code recommendations: GB 50011-2010 ($\text {FAA} = 1 + z/h$, implying $\text {FAA} = 2.0$ at roof level); ASCE 7-16 ($\text {FAA} = 1 + 2z/h$, implying $\text {FAA} = 3.0$); and Eurocode 8 ($\text {FAA} = 1 + 1.5z/h$, implying $\text {FAA} = 2.5$).

**Table 3:** The FAA values at the top of the three structures at different values of $R_\mu$.

Structure Type	Stories	Direction	Fundamental Period (s)	FAA at Roof					
				$R_u= 0.5$	$R_u = 1.0$	$R_u= 1.5$	$R_u= 2.0$	$R_u= 2.5$	$R_u= 3.0$
Reinforced Concrete Frame	5	L	0.66	2.81	2.26	2.03	1.85	1.71	1.36
		T	0.64	2.85	2.28	1.95	1.85	1.70	1.38
	10	L	1.37	2.12	2.07	1.67	1.44	0.96	–
		T	1.31	1.98	2.01	1.64	1.45	1.49	–
	15	L	2.04	1.97	1.65	1.43	1.44	1.42	1.16
		T	1.93	1.96	1.61	1.39	1.43	1.44	1.17
Steel Frame	5	L	0.78	3.30	2.95	2.86	2.77	2.68	2.55
		T	0.90	3.73	3.45	3.19	2.90	2.62	2.40
	10	L	1.65	3.39	3.06	2.81	2.70	2.56	2.39
		T	1.87	2.92	2.74	2.51	2.40	2.31	2.23
	15	L	2.57	2.25	2.04	1.88	1.80	1.75	1.72
		T	2.69	2.02	1.88	1.77	1.74	1.73	1.72
Steel-Concrete Composite Structure	5	L	0.86	3.13	2.27	1.97	1.88	1.70	1.68
		T	0.66	3.20	2.65	2.20	2.05	1.97	1.84
	10	L	1.86	1.80	1.52	1.42	1.37	1.32	1.27
		T	1.38	2.13	1.81	1.71	1.57	1.48	1.44
	15	L	2.89	1.70	1.68	1.62	1.53	1.45	1.38
		T	2.12	1.92	1.71	1.62	1.60	1.62	1.65

“–” indicates the collapse of the main structure; L represents the longitudinal direction and T means the transverse direction.

RC frames exhibit pronounced acceleration amplification at low values of $R_\mu$. For instance, in the 5-storey structure at $R_\mu =0.5$, FAA at roof reaches 2.81 (longitudinal) and 2.85 (transverse), exceeding the GB 50011 recommendation (2.0) by 40.5% and Eurocode 8 (2.5) by 14%, while approaching the ASCE 7–16 upper limit (3.0). As $R_\mu$ increases to 2.0, FAA reduces markedly to 1.44–1.85, generally falling below Eurocode 8 limits yet remaining higher than GB 50011 values. This suggests potential underestimation by the Chinese code during elastoplastic stages.

Steel frames demonstrate consistently elevated roof-level FAA. At $R_\mu =0.5$, the 5-storey steel frame records FAA up to 3.50 (longitudinal), surpassing all code recommendations. Even at $R_\mu =1.5$–2.0, 10- and 15-storey structures maintain FAA values of 2.5–2.9, exceeding GB 50011 and Eurocode 8 thresholds in most cases and approaching ASCE 7-16’s 3.0 limit. These results indicate significant acceleration amplification in upper levels of steel systems after plastic deformation.

Steel-concrete composite structures exhibit moderate responses. The 5-storey composite structure reaches FAA=3.13 (longitudinal) at $R_\mu =0.5$, slightly exceeding ASCE’s limit. However, at medium-to-high value of $R_\mu$ ($R_\mu =2.0$), FAA values for all heights fall below 2.0. Notably, 10- and 15-storey composite structures achieve FAA=1.3–1.7 at $R_\mu =2.0$–3.0, substantially lower than Eurocode 8 and ASCE recommendations and even below GB 50011’s 2.0. This highlights the composite system’s superior control of roof accelerations due to enhanced ductility and energy dissipation capacity.

**Fig. 10:**
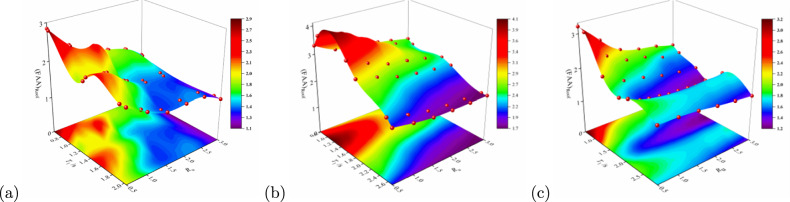
The relationship between FAA and basic period $T_1$ and $R_\mu$ at the roof of three structural types: (**a**) RC frame; (**b**) steel frame, and (**c**) steel-concrete composite structure.

Figure 10 illustrates the three-dimensional variation of roof FAA with $T_1$ and $R_\mu$ across structural types. Key observations include: (1) Peak FAA occurs under low-period (rigid structures) and low-$R_\mu$ conditions, significantly exceeding all code recommendations; (2) Composite structures in high-period/high-$R_\mu$ regions achieve optimal FAA (1.3–1.8); (3) A monotonic decreasing trend in FAA emerges with increasing $T_1$ and $R_\mu$, though response surface gradients and peak locations vary substantially among structural typologies.

### Establishment of the empirical FAA model

#### Relationship model between FAA and $R_\mu$, $T_1$, $z/h$, and structural type

Previous researches^14,26,27^ demonstrate that the FAA coefficient constitutes a nonlinearly coupled function of multiple structural and seismic parameters, exhibiting significant spatial variability and response modulation effects. To develop a broadly applicable FAA prediction model accommodating diverse structural typologies and ductility levels, this study establishes a fitting model incorporating $T_1$, $R_\mu$, and *z*/*h*, based on extensive Nonlinear IDA datasets comprising 4410 floor-level data points. The model is expressed as follows:

7 $$\begin{aligned} \textrm{FAA} = a_0 + a_1 T_1 + a_2 R_\mu + a_3 \left( \frac{z}{h} \right) + a_4 T_1^2 + a_5 T_1 R_\mu + a_6 T_1 \left( \frac{z}{h} \right) + a_7 R_\mu ^2 + a_8 R_\mu \left( \frac{z}{h} \right) + a_9 \left( \frac{z}{h} \right) ^2 \end{aligned}$$

where $T_1$ denotes the fundamental period of the structure (unit: s), $R_\mu$ is the structural strength ratio (i.e. ductility demand, dimensionless), $h$ is the total height of the building, and $z$ is the floor height above the base. Coefficients $a_0, a_1, \ldots , a_9$ are the regression parameters, which vary by structural type. Table 4 summarises the regression coefficients for different structural types.

**Table 4:** The fitting results of parameters.

Structure type	Parameters										$R^2$
	$a_0$	$a_1$	$a_2$	$a_3$	$a_4$	$a_5$	$a_6$	$a_7$	$a_8$	$a_9$	
Concrete Frame	0.73	0.23	−0.13	2.50	−0.02	0.06	−0.70	0.02	−0.36	−0.28	0.73
Steel Frame	0.70	0.43	−0.17	3.24	−0.13	0.07	−0.77	0.02	−0.28	0.14	0.90
Steel-Concrete Composite structure	1.31	−0.28	−0.36	1.83	0.08	0.07	−0.48	0.07	−0.27	0.30	0.80

The model is formulated based on structural dynamic response mechanisms, systematically incorporating primary influencing factors, interactive couplings between variables, and higher-order nonlinear terms to account for three control mechanisms: (1) *Primary factor terms* (e.g., $T_1$, $R_\mu$, *z*/*h*) capture structural frequency characteristics, nonlinear energy dissipation capacity, and modal response intensity at different floors—key drivers of FAA variation. Here, $T_1$ quantifies structural sensitivity to seismic input frequencies, $R_\mu$ represents ductility demands in nonlinear phases, and *z*/*h* reflects acceleration amplification from higher-mode participation in upper storeys. (2) *Cross-coupling terms* (e.g., $T_1 \cdot R_\mu$, $T_1 \cdot z/h$, $R_\mu \cdot z/h$) address coupled response effects, capturing nonlinear FAA growth under combined nonlinearity and modal interactions. These terms embody synergistic modulation between input spectrum properties and structural hysteretic behaviour. (3) *Higher-order nonlinear terms* (e.g., $T_1^2$, $R_\mu ^2$, $(z/h)^2$) enhance peak response prediction capability under strong seismic excitations, particularly for high-rise structures.

Theoretically, this model extends the “*z*/*h*–$R_\mu$” framework proposed by Surana et al. by introducing period-modulation mechanisms and structural typology variations, significantly improving fitting accuracy and physical interpretability. As Table 4 demonstrates, the model achieves high goodness-of-fit across structural systems: RC frames ($R^2 = 0.73$), steel frames ($R^2 = 0.90$), and steel-concrete composite structures ($R^2 = 0.80$) exhibit robust statistical compatibility and engineering applicability.

Calibrated for $R_\mu \in [0.5, 3.5]$ and $T_1 \in [0.6\,\text {s}, 3.0\,\text {s}]$, the model applies to RC frames, steel frames, and steel-concrete composites. It reliably predicts floor-wise acceleration amplification during earthquakes and provides a validated basis for refining floor response spectra (FRS) and component amplification factors (CAF) in engineering practice.

## FAA model verification and validation

To evaluate the applicability and predictive accuracy of the FAA model proposed in this study (Eq. 7), a systematic comparison is conducted against existing design codes^4,6^ and two representative empirical models^15,16^. The comparison includes the following components: (1) use of a unified set of input parameters ($z/h$, $T_1$, structural type); (2) adoption of a measured dataset consisting of 59 instrumented buildings collected from the CESMD database; (3) calculation and comparison of prediction error metrics, including Mean Squared Error (MSE), Mean Absolute Error (MAE), and the coefficient of determination ($R^2$); and (4) theoretical evaluation of model construction mechanisms and applicable domains.

### Comparison with code-based models

To assess the accuracy of the FAA equation derived from Incremental Dynamic Analysis (IDA), measured FAA values for 59 buildings—compiled by^12^ from the CESMD database—are used as reference. As most of these buildings experienced ground motions within the elastic range, their $R_\mu$ is assumed to be 0.5. While this dataset primarily validates the model’s performance for elastic responses, the proposed FAA model itself is derived from extensive nonlinear IDA simulations, ensuring its applicability to structures subjected to significant nonlinear behaviour as well. Predicted FAA values are computed using the proposed model and then compared with measured values. Prediction errors are evaluated using the Mean Squared Error (MSE), Mean Absolute Error (MAE), and the coefficient of determination ($R^2$).

According to the error metrics shown in Table 5, the proposed FAA model consistently outperforms the others across all evaluation indices. Notably, the coefficient of determination ($R^2$) is positive, indicating that the model explains a meaningful portion of the variance in the observed data.

**Fig. 11:**
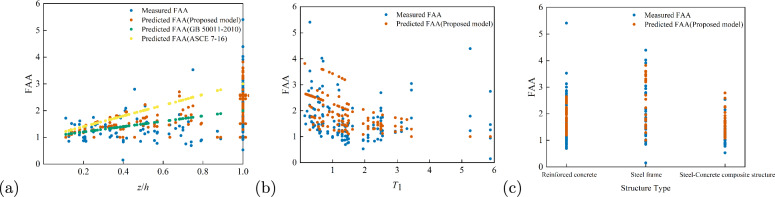
Comparison of the FAA Model Proposed in This Paper with FAA Models under Different Standards: (**a**) FAA under different values of *z*/*h*; (**b**) FAA under different values of $T_1$, and (**c**) FAA under different structural types.

As shown in Fig. 11a, the FAA values of the 59 buildings from the CESMD database analysed by Anajafi et al. exhibit a wide distribution across the entire $z/h$ range. The predictions from the proposed FAA model are mainly concentrated in the range of $z/h = 0.2$ to $0.6$, where a fair degree of agreement with the measured data is observed. However, the model shows noticeable deviations in the lower $z/h$ region. Compared with the proposed model, the^6^ model yields more clustered predictions, but significantly underestimates FAA values when $z/h$ increases. In contrast, the^4^ model presents a more scattered distribution and tends to overestimate FAA when $z/h$ is large. Although different models demonstrate varying trends in the relationship between $z/h$ and FAA, the^6^ model and the proposed model show relatively better consistency with the observed data. From Figure 11b, the relationship between the $T_1$ and FAA indicates that FAA values for the 59 buildings are broadly distributed across the $T_1$ domain, particularly densely within the range of $T_1 = 1.0$ to $2.0\,\textrm{s}$. The proposed FAA model shows better predictive accuracy for smaller values of $T_1$, but deviations become significant when $T_1$ exceeds 2.0 s. As shown in Fig. 11c, the FAA values are distributed across various structural types, especially in RC and steel–concrete composite buildings. The predictions of the proposed FAA model are relatively concentrated across structural types and show good agreement with measured values for composite structures. However, the model tends to underestimate FAA values for steel frame buildings.

To quantitatively evaluate model accuracy, the Mean Squared Error (MSE), Mean Absolute Error (MAE), and coefficient of determination ($R^2$) are computed for the proposed model and compared with the GB 50011-2010 and ASCE 7-16 models. These error indicators are computed over the full dataset and reflect the overall performance of each model across all samples rather than any single figure. MSE is defined as the mean of the squared differences between predicted and measured values, while MAE is the mean of the absolute differences. The formulas are presented in Equations (8):

8 $$\begin{aligned} \textrm{MSE} = \frac{1}{n} \sum _{i=1}^{n} (y_i - {\hat{y}}_i)^2,\qquad \textrm{MAE} = \frac{1}{n} \sum _{i=1}^{n} |y_i - {\hat{y}}_i| \end{aligned}$$

where $y_i$ and ${\hat{y}}_i$ represent the measured and predicted FAA values, respectively, and $n$ is the number of samples. where $n$ is the total number of samples, $y_i$ denotes the measured FAA value of the $i$-th sample, and ${\hat{y}}_i$ is the corresponding predicted value.

Both MSE and MAE are used to evaluate model prediction errors. MSE is more sensitive to large deviations due to squaring the error term, whereas MAE is less influenced by large errors. By computing both indices, it is possible to compare the predictive performance of different models—lower error values indicate better prediction accuracy.

Figure 11 provides a visual representation of model performance, while Table 5 presents a quantitative comparison based on the error metrics. From Table 5, it can be observed that the FAA model proposed in this study achieves the best overall performance, with the lowest MSE and MAE and a positive $R^2$, indicating that the model can explain a reasonable proportion of the variance in the observed data. In contrast, the FAA model in^6^ yields higher MSE and MAE values, with a negative $R^2$, suggesting poor explanatory power with respect to the variance in FAA values. Similarly, the FAA model in^4^ performs the worst, with both large MSE and MAE values and a negative $R^2$, indicating a weak predictive capability.

Overall, the proposed FAA model shows relatively robust performance, particularly when predicting FAA for RC and steel frame structures. In most cases, the model remains stable and reliable. The FAA model in^6^ shows relatively low $R^2$ and exhibits larger errors under certain conditions. The model in^4^ demonstrates the poorest predictive performance among the three. Importantly, the FAA model developed in this study incorporates key influencing parameters, including the *z*/*h* of non-structural components within the building, the nonlinear behaviour of the structure, vibration period, structural type, and strength ratio. In contrast, the FAA expressions in^4,6^ consider only the *z*/*h*, potentially neglecting critical factors influencing floor acceleration response.

**Table 5:** Comparison of Errors and Fit of the Proposed FAA Model with Different Standard FAA Models.

Model name	MSE	MAE	$R^2$
Proposed FAA model	0.50	0.51	0.20
GB 50011-2010	0.68	0.59	−0.08
ASCE 7-16	1.06	0.84	−0.69

### Comparison with other research-based FAA models

**Fig. 12:**
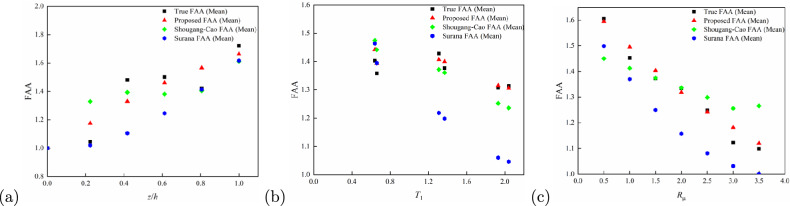
Comparison of the FAA Model Proposed in this paper with the FAA Models of^15,16^: (**a**) FAA under different values of *z*/*h*; (**b**) FAA under different values of $T_1$, and (**c**) FAA under different values of $R_\mu$.

To further validate the broad applicability and accuracy of the proposed model, a comparison was conducted with the FAA models developed by^15^ (Model S) and^16^ (Model C). The procedure is as follows: First, a unified parameter input range was determined, with $z/h$ ranging from 0 to 1, $T_1$ covering both short- and long-period ranges (from 0 to 3.0 s), and $R_\mu$ ranging from 0 to 3.0. Then, FAA predictions were calculated for the proposed model, Model S, and Model C based on the above parameter ranges. Simultaneously, “true FAA values” (True FAA) were obtained through dynamic time history analysis. In the time history analysis, the material and geometric parameters of the structures strictly followed the values given in section “Original model design” (e.g., concrete grade and reinforcement type for RC frames, steel material grades for steel frames, and steel-concrete composite parameters for composite structures), while the selected ground motion inputs were representative records from section “Ground motion selection and IDA method” to ensure the reliability of the simulation results. Finally, the prediction results from the three models were compared with the True FAA values, and the comparison results are presented in Fig. 12a–c and Table 6.

From Figure 12a (FAA comparison at different $z/h$ values), it can be observed that throughout the entire vertical distribution range of $z/h$ from 0 to 1, the predictions from the proposed model closely align with the simulated True FAA values. Specifically, over the entire height range, the predictions from Model S significantly underestimate the True FAA values. In contrast, Model C overestimates the FAA at $z/h \approx 0.2$ (lower floor region), exhibiting an excessive amplification trend. In comparison, the predictions from the proposed model are smoother across the entire $z/h$ range and exhibit a more reliable match with the True FAA values.

Regarding the effect of $T_1$ (Fig. 12b), the proposed model demonstrates stable FAA predictions at both small $T_1$ (short-period structures, e.g., $T_1 < 0.5s$) and large $T_1$ (long-period structures, e.g., $T_1> 1.5s$). Model S provides accurate predictions for $T_1$ values below 0.8s but exhibits significant fluctuations in predictions when $T_1$ exceeds 1.2s, indicating limited adaptability to long-period structures. Model C provides good prediction accuracy around $T_1 \approx 1.3-1.4s$, but significantly deviates from the True FAA values for both low-period structures ($T_1 < 0.8s$) and high-period structures ($T_1> 1.6s$). The proposed model effectively captures the nonlinear variation trends in FAA as a function of $T_1$, consistent with the True FAA values.

In the FAA comparison across different $R_\mu$ values (Fig. 12c), the True FAA values decrease as $R_\mu$ increases. The proposed model closely follows this trend, maintaining good consistency with the True FAA values across the entire range of $R_\mu$ from 0 to 4.0. In contrast, Model S significantly underestimates the FAA when $R_\mu$ is large (e.g., $R_\mu \approx 3.5$), reflecting its inability to adequately capture the inelastic energy dissipation effects. The proposed model resolves this issue through the nonlinear ductility adjustment factor (related to $R_\mu$) introduced in section “Establishment of the empirical FAA model”. Model C performs better than Model S, but still shows deviations from the True FAA values at both low $R_\mu$ (e.g., $R_\mu < 0.5$) and high $R_\mu$ (e.g., $R_\mu> 3.0$).

**Table 6:** Comparison of Errors and Fit of the FAA Model Proposed in this paper with the FAA Models of^15,16^ under Varying Parameters.

Parameter Model Name		MSE	MAE	$R^2$
$R_\mu$	Proposed model	0.05	0.18	0.84
	Model C	0.09	0.25	0.73
	Model S	0.14	0.30	0.58
$T_1$	Proposed model	0.04	0.15	0.85
	Model C	0.05	0.19	0.79
	Model S	0.09	0.24	0.63
*z*/*h*	Proposed model	0.05	0.18	0.84
	Model C	0.09	0.24	0.73
	Model S	0.14	0.30	0.58

Table 6 further substantiates the excellent predictive performance of the proposed model from a quantitative perspective: for the key parameters $R_\mu$, $T_1$, and $z/h$, the Mean Squared Error (MSE) and Mean Absolute Error (MAE) of the proposed model are significantly lower than those of Model S and Model C, while the coefficient of determination $R^2$ is notably higher. The proposed model consistently outperforms the other models across all parameter ranges, confirming its ability to provide more accurate and consistent predictions across a wide range of structural and seismic parameters.

Overall, the proposed model integrates the period shift mechanism, nonlinear ductility adjustment factor, and structural type sub-models, overcoming the elastic assumptions of Model S that lead to elastoplastic scenario deviations, and compensating for the limitations of Model C, which is only applicable to RC frames. The proposed model exhibits outstanding accuracy, consistency, and theoretical robustness within the $z/h - T_1 - R_\mu$ response space, reliably capturing floor acceleration amplification behaviours under varying structural and seismic conditions. The prediction distortions or saturation effects of Model S and Model C under extreme parameter conditions further highlight the necessity of incorporating nonlinear and period-dependent effects in FAA models, and validate the advantages of the proposed model for engineering applications.

## Discussion

Although the FAA model proposed in this study achieves multi-parameter coupled predictions, it still has the following limitations due to the constraints of the research boundaries. Future work can focus on optimising the following aspects:

1. The model is applicable only to 5–15 story reinforced concrete (RC) frames, steel frames, and steel-concrete composite frames. It does not include shear wall structures, hybrid structures, or low-rise ($<5$ stories) and super-tall ($>15$ stories) buildings. Future research will include Incremental Dynamic Analysis (IDA) for these structures and introduce a “structural system correction factor” to expand the applicability of the model.
2. The parameter ranges are currently limited to $R_\mu \in [0.5,3.5]$ and $T_1 \in [0.6,3.0\,\text {s}]$, and special site conditions (such as soft soils and liquefaction-prone sites) are not considered. Future work will strengthen the seismic intensity analysis to obtain data for $R_\mu = 4.0 \sim 5.0$, and supplement short-period structure models and soft soil foundation scenarios.
3. The model currently neglects non-structural additional mass, vertical components of ground motion, and construction deviations. Additionally, the representativeness of the verification data is insufficient. Future research will incorporate correction terms for additional mass, conduct cross-software verification (ETABS/SAP2000), and integrate small-scale shaking table tests to further enhance the model’s reliability and generalisability.

## Conclusions

This study investigated the nonlinear seismic responses of multi-storey frame structures and developed a multi-parameter nonlinear model for predicting FAA. IDA was employed to quantify the effects of structural height, fundamental period, strength ratio, and structural type. The main conclusions are summarised as follows:

1. IDA analysis of 5–15-story RC frames, steel frames, and steel-concrete composite frames reveals that Floor Acceleration Amplification (FAA) increases monotonically with relative height $z/h$, reaching a peak at the top floor ($z/h = 1$). FAA significantly decreases (by 40%–60%) as the ductility demand coefficient $R_\mu$ increases from 0.5 to 3.5. As the fundamental period $T_1$ increases, the FAA distribution stabilises. For short-period ($T_1 < 0.8s$) rigid structures, the top-floor FAA can reach up to 3.7, while for long-period ($T_1> 1.5s$) flexible structures, it drops below 2.0. Among the structural types, steel frames exhibit the most stable FAA (fluctuations $< 25\%$), RC frames are the most sensitive to parameters (with a reduction of up to 60%), and composite structures achieve a balanced performance.
2. Based on 4,410 floor data points, a model incorporating the main factors ($z/h$, $T_1$, $R_\mu$), interaction terms (e.g., $T_1 \cdot R_\mu$), and higher-order nonlinear terms (e.g., $T_1^2$) was developed. The coefficients for different structural types were determined: RC frame ($R^2 = 0.73$), steel frame ($R^2 = 0.90$), and composite structure ($R^2 = 0.80$). This model overcomes the limitations of existing codes, which rely solely on $z/h$, and significantly improves the prediction accuracy for strong seismic and long-period scenarios.
3. The proposed FAA model outperforms existing models (GB 50011-2010, ASCE 7-16, Model C, and Model S) in terms of both accuracy and fitting. Compared to GB 50011-2010, the Mean Squared Error (MSE) decreased by 26.5%, and compared to ASCE 7-16, the MSE reduced by 52.8%. Additionally, the proposed model showed lower MSE and Mean Absolute Error (MAE) values and higher $R^2$ than both Model C and Model S, demonstrating superior prediction accuracy and fitting across key parameters such as $R_\mu$, $T_1$, and $z/h$.
4. The model can be directly applied for calculating equivalent seismic forces for ceiling systems, mechanical equipment, etc. In short-period ($T_1 < 0.8s$) and low $R_\mu$ ($R_\mu < 1.0$) scenarios, the model enhances design safety. In long-period ($T_1> 1.5s$) and high $R_\mu$ ($R_\mu> 2.5$) scenarios, it reduces redundancy, providing support for revising the FAA calculation methods in codes.

## Data Availability

The datasets generated during the current study are available from the corresponding author on reasonable request.
